# A Realist Review Protocol into the Contexts and Mechanisms That Enable the Inclusion of Environmental Sustainability Outcomes in the Design of Lean Healthcare Improvement Interventions

**DOI:** 10.3390/ijerph21070868

**Published:** 2024-07-02

**Authors:** Elaine Shelford Mead, Seán Paul Teeling, Martin McNamara

**Affiliations:** 1 UCD Centre for Interdisciplinary Research, Education & Innovation in Health Systems, School of Nursing, Midwifery & Health Systems UCD Health Sciences Centre, D04 VIW8 Dublin, Ireland; 2 Centre for Person-Centered Practice Research Division of Nursing, School of Health Sciences, Queen Margaret University, Queen Margaret University Drive, Musselburgh EH21 6UU, UK

**Keywords:** lean, healthcare, environmental sustainability, environmental waste, realist review

## Abstract

Healthcare makes a significant contribution to the social, economic and environmental benefits of communities. It is correspondingly a significant employer and consumer of both energy and consumables, often at high costs. Lean, a quality improvement methodology focuses on the elimination of non-value add (NVA) activities (steps that do not add value from the perspective of the customer) to improve the flow of people, information or goods. Increasingly, Lean thinking is evolving from its initial focus on eliminating NVA to a more holistic approach that encompasses sustainability. However, little work has been undertaken intentionally, including environmental sustainability outcomes in Lean healthcare interventions. Realist review methodology facilitates an understanding of the extent to which an intervention works, for whom, in what context, why and how, and has proven useful in research relating to Lean interventions in healthcare settings. This protocol provides details for a realist review that will enable an understanding of the specific contexts in which certain mechanisms are activated that enable the inclusion of environmental sustainability outcomes in the design of Lean healthcare improvement interventions.

## 1. Introduction

The current environment for the delivery of healthcare is hugely complex and politicized. This means, for example, that in many countries across Europe, although the average proportion of gross domestic product spent on healthcare, defined as the final consumption of health goods and services, was over 10.9% in 2022 [1], which is significant, the demand for and expectations of health services continue to grow. The current models of the delivery and organization of healthcare developed in the Western world are now regularly described as unsustainable [2], with the largest percentage of funding allocated to hospital-based rather than community-based services. 

The term unsustainable, when related to the delivery of healthcare, is used to describe the consequences of the combination of a complex set of issues, which include an ageing population, an increase in the expectations of patients and their families, and the rising costs from new technologies, procedures and drugs [3]. There is also evidence of wasteful practices and organization of processes within the sector, the lowest available estimates for which exceeded 25% of total healthcare expenditures in the US in 2019 [4]. Current estimates suggest that 15% of the total amount of waste produced in healthcare can be classified as hazardous and can be infectious, toxic or radioactive, which, if not properly treated, forms a potential risk both to human health and to the environment [5]. This continued waste of healthcare resources and generation of healthcare waste affects the ongoing sustainability of the healthcare system.

### 1.1. Sustainability

#### 1.1.1. Defining Sustainability

In the last two decades, there has been an increase in the number of publications relating to sustainability; however, despite this, sustainability remains an open concept with multiple interpretations and meanings that are often context specific [6]. Sustainability has been elusive to define [7] and has been described as an ideology constituting a set of beliefs and values to establish how life might be better organized [8]. Sustainability is a concept that comprises three fundamental categories: environmental sustainability, economic viability and social equity. These three categories are also known as the three pillars of sustainability or the three pillars of sustainable development. These pillars serve as a framework for defining and evaluating sustainable decisions and issues [9]. 

Environmental sustainability refers to the responsible use and management of natural resources to prevent environmental degradation and contamination [10]. The delivery of healthcare and the provision of services to the healthcare system result in environmental stressors, such as greenhouse gas emissions, particulate matter, and air pollutants and compromise scarce water resources across the globe. Economic sustainability refers to the ability of an economy to maintain and improve its standard of living over time. Social sustainability refers to the ability of a society to meet the needs of its citizens while maintaining social cohesion and equity. It has been acknowledged by Lenzen [11] that those people harmed by the environmental footprint of healthcare often live far away from those who benefit from the healthcare provided. Together, the three pillars of sustainability provide a comprehensive framework for evaluating sustainability issues and making sustainable decisions [9]. Gro Harlem Brundtland’s [12] seminal work is widely acknowledged as bringing the term environmental sustainability into policy discourse, calling for ‘‘a new era of economic growth—growth that is forceful and at the same time socially and environmentally sustainable’’. Regarding environmental sustainability, one of the three pillars that make up the totality of a view of sustainability is [6], this protocol paper discusses research with a focus on the impact of healthcare institutions on environmental sustainability, recognizing that economic viability and social equity need to be in alignment with environmental sustainability goals to provide a health system, which will be able to provide for future generations.

#### 1.1.2. Environmental Sustainability in Healthcare

Healthcare accounts for approximately 10% of the global economic output, but there is large variability between countries [13]. Compared to many manufacturing sectors, healthcare has a relatively small carbon footprint. However, it still contributes significantly to greenhouse gas emissions, estimated to be approximately 4.4% globally [13]. Healthcare, in particular hospitals, comprises an energy-intensive sector because of the requirement for continuous operation and services, such as lighting, heating, ventilation and cooling required to create a high-performance clinical environment to support healthcare delivery [14]. As a result, the healthcare industry has been identified as a significant contributor to greenhouse gas emissions that are responsible for climate change, along with other pollutants and unsustainable practices that ultimately have negative impacts on human health and well-being. Considering the healthcare industry’s mission to do no harm, it has an inherent responsibility to reduce its environmental footprint [15].

The World Health Organization in its description of an environmentally sustainable healthcare system [16] declares it ‘as a health system that improves, maintains or restores health, while minimizing negative impacts on the environment and leveraging opportunities to restore and improve it, to the benefit of the health and well-being of current and future generations’. In its translation of this ambition, the Royal College of Physicians more recently acknowledged the vital role that clinicians and other healthcare professionals in the National Health Service (NHS) can play in improving the sustainability of healthcare through changes to practice and the way that care is delivered [17], having previously recognized sustainability as an additional domain of quality in healthcare [18]. The inclusion of environmental sustainability seems a natural extension of the current health system’s focus on economic viability [19] and commitment to social equity. Whilst awareness of the importance of environmental protection is increasing, interest remains limited and without coordinated or intentional action. 

In response to the ongoing challenges of delivering quality, safe and effective care, healthcare staff internationally have been seeking to improve processes through the use of improvement methodologies such as Lean [20]. Lean, a process improvement methodology developed in the Japanese Motor industry [21], is now considered to be one of the most popular process improvement methodologies used in healthcare internationally [20]. 

### 1.2. Lean

Lean was developed for healthcare as an adaptation of the method of industry pioneers in quality improvement, the Toyota Production System, which differed from the principles of mass manufacturing in the automotive industry through the way that they engaged the workforce to initiate improvements rather than simply responding to problems [22].

The approach has been in use in healthcare since 2001 in the UK and since 2002 in the USA and has become one of the most popular process improvement methodologies used in healthcare [23,24,25]. This translation and adaptation was successful in some highly effective healthcare organizations with leaders committed to change [26,27] but has not been successful in all cases. 

Lean in healthcare can be seen as a set of operating methods and a philosophy, which creates the maximum value as defined by the customer, primarily the patient but also those involved within the delivery or organization of services [28]. Whilst some authors view Lean as simply a toolkit within a methodology [29,30] other authors disagree with this narrow description [20,25,31,32], and suggest that Lean should be viewed as a philosophy, which is only successfully implemented if it fully engages people to improve processes [24,33]. It is this commitment to Lean as a philosophical approach in tandem with a focus on people and processes, particularly in hospital-based settings [34], that appears to enable an organization to become ‘Lean’. As one of several recognized and practiced quality improvement approaches within healthcare, Lean has been shown to have benefits when applied to this complex system [34]. Healthcare organizations that have adopted Lean give form to the idea of constantly striving for continual improvement [35,36], although studies show that implementation may be isolated rather than system-wide [24,37].

In acknowledging Lean as an established methodology and philosophy for improvement in healthcare, it is valid to consider it as a potential vehicle for other initiatives, such as engagement around the sustainability agenda to reduce the carbon footprint of the business [38]. The healthcare sector, with its reliance on fossil fuels to energize buildings, was responsible for 4–6% of global greenhouse gas emissions in 2017 [39] and is now taking early but important steps to reduce its emissions. However, it lacks a cohesive and reproducible whole-system approach for engagement. Lean may be a solution to this problem [38].

Although uniting the removal of waste from healthcare systems as an improvement methodology together with the active work of the sustainability teams appears intuitively the right thing to do, there is little evidence in the literature of this being a consistent approach across businesses and no evidence of this approach being taken intentionally to scale across healthcare, although there have been some attempts to validate the combination of Lean and organizational sustainability efforts approach at a clinically specific level [40,41].

### 1.3. Sustainability Goals within Lean Interventions

Hackbarth and Berwick [42] describe six categories of healthcare waste: overtreatment, failures of care coordination, failures in execution of care processes, administrative complexity, pricing failures and fraud and abuse. It has been acknowledged that approximately one-fifth of the cost of healthcare can be described as wasteful, providing no or minimal impact towards good health [4,43]. Whilst the language of fraud and abuse may not resonate directly with the seven Lean wastes originally developed by Taiichi Ohno, Chief Engineer from Toyota, as defects, overproduction, waiting, transport, inventory, motion and excess processing [44], it is possible to recognize the overlaps with the six categories previously described by Hackbarth and Berwick [42].

Healthcare has adapted Lean tools and techniques from across the most effective businesses to remove these wastes, focusing on both clinical and non-clinical pathways. Quality improvement, and specifically Lean methodology, has been adapted and applied to healthcare by pioneer organizations in North America [26,27]. Lean is a way to reduce waste or non-value-added activities from processes and add value for stakeholders, in this particular case both patients and staff. It can be described as reducing the overburdening of staff by streamlining the processes to be more efficient. Waste reduction in this context is often measured in terms of time saved, quality improved, better patient and staff experiences of care and financial benefits through the application of rigorous tools and techniques. In addition to the work of Ohno, an eighth important category was included in the 1990s when the Toyota Production System was adopted in the Western world: the waste of human effort. Not having the opportunity to practice to the full scope of their potential or license immediately resonates with many healthcare workers and is a critical element of many Lean initiatives. 

To date, in the application of Lean in healthcare, there has been little consideration provided to the development of metrics to measure the waste of natural resources, such as water or energy, nor to the environmental impacts of the redesign and delivery of care pathways. For example, whilst teams may redesign processes to reduce the number of items being used, little attention has been provided, thus far, to the consideration of the manufactured source of the specific item or even the current impact of the disposal of the item. This becomes important when it is recognized that 62% of the total healthcare emissions arise from the supply chain, estates and facilities, pharmaceuticals and medical devices and travel [45]. The removal of waste in healthcare means very different things to different groups, with clinicians involved in pathway improvements looking at efficiencies and streamlining and professional sustainability experts looking at the continuous reduction in the use of natural resources or the disposal of clinical waste. There is a discrete overlap in these two perspectives when both groups consider, for example, the single use of plastic items and packaging. Both approaches would seek to reduce the number of items used, one from a cost and time to utilize perspective [46] and the other from an impact of manufacturing, cost of transportation and disposal perspective. 

Healthcare, in particular hospitals, along with the consumables that are made and transported for their use in clinical care, is responsible for 5% of the UK’s carbon footprint, with the figure being higher in some countries [47]. Hospital buildings must be heated and ventilated 24 h a day, seven days a week; they are energy-hungry and, if the contribution of the manufacturing that serves the healthcare industry is included, they account for a significant impact on the global environment due to their emissions [14]. 

Increasingly, healthcare organizations across Europe are mandated to commit to a reduction in their carbon impact and have environmental sustainability as a key deliverable at the board level [45]. The responsibility for the delivery of this agenda is often undertaken by Estates and Support Service teams who are increasingly expanding their expertise through the appointment of environmental sustainability experts to lead the work.

Looking more widely across other business systems, Zokaei et al., [48] argue that there is a “logical fit between economic and environmental waste reduction”, between what they describe as Lean and Green, with “Lean as a descriptor of all forms of quality improvement activities” and “Green to capture all management practices aimed at improving the environmental performance of the firm”. In Lean systems, waste is any activity that does not add value to the customer, while ‘Green’ concerns the inefficient use of natural resources. As Garza-Reyes [49] puts it “For the lean management philosophy, waste refers to any activity that does not add value to the product while for the green concept, waste is related to the wasteful consumption of water, energy or any natural resource.”

Salvador et al. [50] adapted the work of Dües [51] to illustrate the relationship between a Lean and Green approach in business, which can be further explored in healthcare. They purport that an integrated approach seems to be an easier and better option on the path to Leaner and greener business practices. The overlap with the organization and delivery of healthcare in some areas of product planning and design, supply chain and quality management, organizational culture and performance and logistics gives rise to an opportunity that could be adapted to be more specific to healthcare. It is this opportunity that will be explored in this research. 

### 1.4. Methodology

The degree to which environmental sustainability is integrated into Lean improvement interventions may vary, and the field is evolving [52]. The inclusion of organizational environmental sustainability goals specifically into Lean healthcare is under-reported in the literature [53]. There is no clear understanding of the contexts in which Lean interventions that include environmental sustainability outcomes occur, nor of the mechanisms that encourage engagement in those interventions that lead to specific anticipated environmental sustainability outcomes. A realist approach can be used to examine these contexts, mechanisms and outcomes. 

Realist inquiry is a research approach that includes two main methods: realist review and realist evaluation. Realist review is also known as realist synthesis and involves analyzing already existing data, including stakeholders’ views and opinions [54]. Realist evaluation is used for primary research and involves collecting data directly from the source [55]. The focus of realist inquiry is on interventions and there is a growing body of work that uses realist inquiry to analyze interventions in healthcare organizations [56,57], such as those using Lean methodology [25,58].

Research strategies such as systematic reviews look for the answer to the question ‘what works?’. In a realist inquiry, there is a focus not only on ‘what works’ but also on ‘what works for whom, why it works and in what circumstances’ [59]. Realist review goes beyond traditional systematic reviews by focusing on the underlying theories and mechanisms that explain how interventions work in specific contexts. It is a robust approach that recognizes and embraces the complexity of social systems [59]. Realist review acknowledges that theories cannot and do not always offer explanations or predict outcomes in every context, for example, in patient safety programs [60]. A realist review encompasses reviews of existing studies that use a wide range of research and evaluation approaches and have no particular bias towards either quantitative or qualitative methods. Wong, et al. [61] see realist reviews in the context of the ‘what works, for whom, in what circumstances’ approach as being non-judgmental and explanatory and, whilst borrowing some ideas from traditional systematic reviews, they are more iterative, testing and building theory.

The underlying focus of a realist review recognizes the explanatory power and contribution to the knowledge of ‘generative causation’ through the first principles of Context Mechanism Outcome configurations CMOcs [62]. CMOcs comprise the Context (C) that denotes a wide range of conditions that affect any programme. The variation in response of individuals to the program will be dependent on factors such as their understanding of what they can do and what they need to contribute [62]; this is an example of a Mechanism (M). The hypothesis as to a program’s Outcomes (Os) concerns the program’s results. The Context, Mechanism and Outcome are often expressed in the formula C + M = O (CMO), with configurations (c) of multiple CMOs generating the term CMOcs. A realist review will facilitate an understanding of how Lean interventions that include environmental sustainability outcomes in their design interact with specific contexts to trigger particular mechanisms that lead to observable anticipated outcomes. We now elaborate on the use of realist review to address the research questions posed by this study.

## 2. Materials and Methods

### 2.1. Aim

The realist review outlined in this protocol aims to understand the use of the term environmental sustainability in a healthcare context and to develop a clear narrative regarding the specific relationship between Lean healthcare improvement interventions and environmental sustainability. It aims to explore, describe and understand barriers to achieving environmental sustainability and the contexts and mechanisms that may support and enable local teams to deliver more environmentally sustainable outcomes from their Lean improvement interventions. 

### 2.2. Research Questions

A realist review protocol does not provide answers to the research questions; rather, it serves as a guide for conducting a subsequent realist review of the literature. This approach enables the research questions to be systematically addressed through an iterative and theory-driven process [61]. The purpose of the protocol is to structure the review, ensuring that it explores how and why interventions work in specific contexts, thus facilitating the eventual answering of the research questions [59]. The realist review described in this protocol will be guided by these research questions:

- What contextual factors facilitate the design of Lean healthcare improvement interventions that include environmental sustainability outcomes, and what mechanisms enable their inclusion?
- What contextual factors facilitate the deployment of Lean healthcare improvement interventions that include environmental sustainability outcomes in their design, and what mechanisms enable the engagement of stakeholders with these interventions, resulting in the achievement of the anticipated outcomes?

### 2.3. Stages of the Realist Review

A realist review involves the analysis and interpretation of existing data. In essence, it is the application of the realist approach to retrospective literature reviews [54], acknowledging that theories do not and cannot always offer explanations or predict outcomes in every context [60]. Based on Pawson’s [63] work, and the interpretation of that work by Velonis et al. [64], we can identify a five-step approach to a realist review as follows:

1. Agree on the scope of the review and identify hypotheses that will explain mechanisms that are causative factors in change.
2. Identify a starting point to search for evidence.
3. Review primary studies and retrieve data.
4. Synthesize evidence and develop conclusions.
5. Refine theory iteratively and disseminate findings.

**Figure 1:**
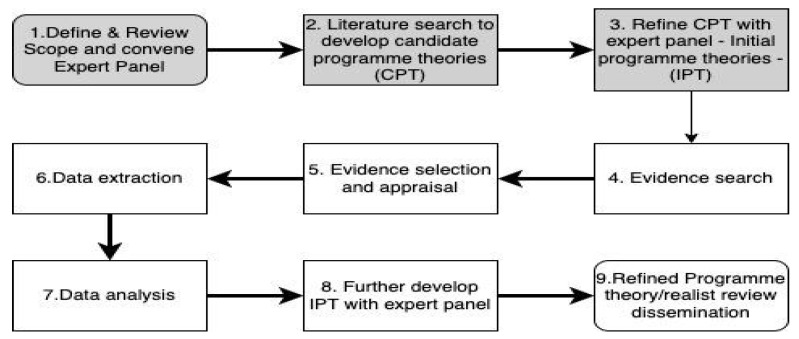
Review Design.

These steps are congruent with RAMESES (Realist And Meta-narrative Evidence Syntheses: Evolving Standards) [65]. The RAMESES guidelines are widely regarded as the benchmark for conducting realist reviews, providing comprehensive standards and resources. They offer structured guidance on methodology and reporting practices essential for ensuring rigor and transparency in realist reviews [61,65]. Figure 1 outlines the steps we will undertake to complete the realist review according to the RAMESES guidelines. Steps 1–3 have already been completed to inform this realist review protocol.

#### 2.3.1. Scope of the Review and Expert Panel Formation 

Defining the scope of any realist review question can prove challenging, given the application of this methodology to complex systems like healthcare [66]. The scope of this review concerns the inclusion of environmental sustainability outcomes in Lean improvement interventions in healthcare settings. Given the limited research into Lean interventions with environmental sustainability goals in healthcare [38], the scope of the review will consider the inclusion of such goals in the design of Lean improvement initiatives in non-healthcare settings. This will allow for the realist review to take account of the transferability of relevant review findings from sectors other than healthcare to health and social care settings. Generally, realist reviews commence with the development of one or more candidate program theories (CPTs) (hypotheses) and end with a more developed theory [25]. The first step in conducting a realist review is therefore to develop CPTs that seek to explain how the designers and implementers of Lean improvement interventions with stated environmental sustainability outcomes expect them to work, and why [62]. CPTs do this by describing the context, mechanism, and outcomes at play [66]. 

A realist review is theory-led because it consults the literature and relevant stakeholders to develop and test theories. The inclusion of stakeholders and explicit, extensive, iterative engagement with them grounds the review in local contexts to facilitate theory refinement [67]. To inform CPT development for this review protocol, a stakeholder consultation was undertaken with leadership teams and improvement specialists within organizations that had or were in the process of including environmental sustainability outcomes in their Lean improvement training and interventions [68].

The use of expert panels, a group of individuals who are judged to possess expertise and experience relevant to the research topic or intervention under investigation, guides the development of the initial program theory and assists in iterative theory development [69]. An engaged expert panel provides expertise and validates program theories iteratively, ensuring rigor [70]. For this study, a panel has been convened consisting of acknowledged subject experts with a particular interest in the application of Lean methods in healthcare [18,71], the environmental effect of healthcare through its organization and delivery, and the impact on the engagement and practice of individuals [72,73]. The panel’s role is to provide feedback, challenge findings, identify gaps in current knowledge and provide further insights into the information already collected to determine whether and which theories merit further development and testing. In addition, the ability of the expert panel to interpret realist review results and develop additional lines of inquiry as program theories are recognized as an additional value of their engagement [70]. 

#### 2.3.2. Literature Search to Develop Candidate Program Theories (CPTs)

Pawson and Tilley [62] emphasize the importance of CPT generation at the outset of the evaluation [74]. Before undertaking a realist review, it is necessary to conduct an initial scoping of the literature [75]. A preliminary background search in key databases was undertaken searching article titles, abstracts, keywords, and subject headings to guide the development of the CPTs. The databases CINAHL, EBSCOhost, ProQuest, MEDLINE, PubMed, BMJ best practice, EMBASE, Web of Science, Green FILE, Scopus, ScienceDirect Journals, CAB Direct, JSTOR and Google Scholar were used to identify studies that were relevant to the review questions and involved Lean and environmental sustainability outcomes in healthcare. The searches included combinations of the keywords Health, Healthcare, Lean, Lean Methodology, Green, Environmental Sustainability, and Sustainability. The studies encompassed both empirical and conceptual work. As the theories to be developed were at this stage candidate or ‘rough’ [66], sources of grey literature, editorials, reports, and the websites of relevant organizations, such as The Health Foundation, Healthcare without Harm, the Institute for Healthcare Improvement and Toyota Environmental Challenge 2050 were also included. 

Searches yielded a small number of articles (*n* = 7) directly and fully related to the research questions. The expert panel suggested further areas for consideration for inclusion, specifically websites. An expert panel can provide valuable expertise in relation to sources, including the grey literature (e.g., government documents, reports and websites) that may be relevant to program theory development [76]. From the consultations with stakeholders and the exploratory review of the literature, six CPTs were developed, which were further refined after discussion among members of the research team. This resulted in the development of seven CPTs to be discussed with the expert panel (Supplementary File S1). 

#### 2.3.3. Refine CPTs with the Expert Panel

Within realist review, much of the time and effort needed is spent on developing program theory and developing, confirming, refuting or refining aspects of it [69]. The convened expert panel was adjudicated upon the program theories being investigated. This facilitated an emerging understanding of how and why particular interventions work or do not work in specific contexts by examining the underlying mechanisms activated in those contexts that influence the interventions’ outcomes [77].

In relation to the seven CPTs presented to them (Supplementary File S1), the panel’s adjudication and deliberation led to the following:

- Two CPTs (numbers 4 and 5) were deemed as being aligned with the available evidence and existing understanding in the field and therefore, required no refinement (CPTs confirmed).
- Four CPTs (numbers 1, 3, 6 and 7) were seen as valid in their candidate form but were identified as having incomplete elements of theory, and additional mechanisms, contexts and potential outcomes were identified (CPTs refined).
- One CPT (number 2) was seen as not relevant to the research (CPT refuted).

This consultation and feedback on the CPTs ensures that they have wider validity and consistency with the panel members’ experiences and that nothing crucial was missed [25]. The expert panel review, through adjudication of the presented CPTs, facilitated the development of eight Initial Program Theories (Supplementary File S2).

The expert panel, throughout the research process, will continue to provide ongoing input, expertise and guidance in adjudicating upon the iteratively developed program theory. Having completed steps 1–3 (Figure 1) to inform this realist review protocol, we now set out our planned next steps. 

#### 2.3.4. Evidence Search

The next step will be an evidence search based on keywords derived from the Initial Programme Theories (IPTs) elicited from steps 1 to 3 [78]. PRISMA (Preferred Reporting Items for Systematic Reviews and Meta-Analyses) guidelines are specifically designed for systematic reviews and meta-analyses, providing a structured approach to synthesizing evidence from various study designs, including observational and interventional trials [79]. Realist reviews, which aim to understand complex interventions and contexts rather than evaluate efficacy through controlled trials, benefit from PRISMA’s flexibility in reporting diverse types of evidence and its emphasis on transparency and reproducibility in data synthesis [67]. Searches will be reported in line with PRISMA guidelines for systematic reviews [79] and congruent with RAMESES guidelines [65]. The internet search engines and electronic databases used to develop the CPT (Section 2.3.2) will be used to carry out an evidence search using keywords based on the IPTs identified in steps 1–3 (Supplementary File S2). This search will be augmented by a search of:

- Any literature citing the included papers;
- The literature cited in the reference lists of included papers;
- Grey literature and websites of relevant organizations;
- Any further suggestions from the expert panel.

We will make use of the population-intervention-comparison-outcome-context (PICOC) tool, commonly used in literature reviews, to outline inclusion and exclusion criteria [80,81,82]. The PICOC (Table 1) outlines the current inclusion and exclusion criteria, generated following the development of the IPTs, that will inform the realist review. We recognize that the inclusion and exclusion criteria may be refined and further iterative searches may be needed as the review progresses [59]. The PICOC has been shown to ensure that the selection of items for inclusion is systematic, consistent, and independent of factors such as sample size or funding source that may affect the direction of the inquiry or the interpretation of results [82].

The search strategy reflects a realist review methodology that does not exclude gray literature, which can illuminate causal factors [83]. In line with the realist review methodology, iterative and purposive searches may be necessitated by the need to gather more supporting evidence to develop and test the developing IPTs. This may be to search for further evidence or wider theories that may explain findings and assist in theory refinement [84]. The need for further searches, search terms and strategies will be identified as the review progresses. Search results from electronic databases and other sources will be imported into reference management software and duplicates removed.

#### 2.3.5. Evidence Selection and Appraisal

Retrieved documents will be selected based on their relevance to the research questions (Section 2.2) and on their contribution to the development of IPTs [59]. Brennan et al. [85] advise that when seeking to inform the program theory, the reviewer should be cognizant that even small sections of a primary study may be relevant and contribute to testing a specific hypothesis about the relationships between context, mechanisms and outcomes. Retrieved documents will contribute to a high-quality collection of papers for a theory-driven realist review [37,86] that will further inform and refine the developed IPTs (Supplementary File S2).

The selection of studies for analysis and synthesis will depend on their relevance and rigor. For a realist review, relevance refers to whether a study can contribute to building or testing a program theory, while rigor refers to the credibility and trustworthiness of the methods used to collect the relevant data [78,87]. These criteria will be used to determine whether the literature contributes to testing or developing the IPT and to inform its inclusion or exclusion in the realist review [88]. 

#### 2.3.6. Data Extraction

Data extraction will be a three-step process. Firstly, an initial screening of identified papers by title and abstract, followed by a full-text retrieval and finally appraisal. Initial screening by title and abstract will be undertaken in duplicate by authors 1 and 2. Each title and abstract retrieved from the searches will be reviewed using the inclusion and exclusion criteria (Table 1) to determine suitability. Authors 1 and 2 will screen documents for initial relevance, while Author 3 will further assess the included documents for richness and rigor. Any queries or disparities will be discussed by all three authors. Throughout the entire process, the authors will continue to adhere to the criteria outlined in the RAMESES guidelines [65].

To facilitate the data extraction process, congruent with realist review guidelines, the IPT being ‘tested’ through the realist review will first be rendered apparent through the development and use of bespoke data extraction forms [89,90]. The bespoke nature of the data extraction forms recognizes that data extraction forms in realist reviews will vary based on the underlying theoretical framework, making each form unique to the specific realist review [63,84]. Given the theoretical framework, standardized data extraction forms may be unsuitable, and this is reflected in the design of tailored forms within realist review [66]. These data extraction forms will be used to extract and review information relevant to IPT refinement through the identification of contextual factors, mechanisms and outcomes relating to the research questions.

#### 2.3.7. Data Analysis

Following data extraction, we will import the selected papers into the specialized qualitative software NVivo [91] version 14 (Mac), which has been used in realist reviews [37,92] to facilitate coding and thematic analysis of the data to identify CMOcs. NVivo is highly regarded for its ability to efficiently manage and analyze qualitative data, making it an excellent choice for realist reviews. It supports systematic coding and thematic analysis, crucial for identifying patterns and refining theories iteratively throughout the review process [92]. Where relevant, stakeholders will help us improve the final theoretical framework by sharing their knowledge and expertise in the field. The authors will revisit any stage of the review process as necessary to ensure that we have gathered enough data and reached a state of ‘theory saturation’ [93,94,95].

#### 2.3.8. Further Refine IPTs 

The refined IPTs from the realist review will be presented to and adjudicated by the expert panel, which will provide further feedback on the IPTs to ensure an appropriate interpretation of results [70]. The involvement of the expert panel at this stage will contribute to ensuring that the refined IPTs are robust, contextually sensitive, and reflective of the complexities inherent in the intervention’s implementation and outcomes [25,67]. 

## 3. Dissemination of Findings

The dissemination of the results of the realist review outlined in this protocol will be consistent with the RAMESES reporting guidelines [65]. The intention is to disseminate the findings through the publication of the realist review in a peer-reviewed journal, through relevant conference presentations attended by healthcare, industry, and academic audiences, and for further dissemination to take place through stakeholder involvement in further theory refinement. The realist review will also constitute part of a PhD thesis. The findings of the realist review will inform the next stage of this research, which will be a realist evaluation of the findings with stakeholders involved in Lean healthcare interventions that include sustainability outcomes in their design. This realist review protocol will be registered with PROSPERO.

## 4. Discussion

The integration of environmental sustainability outcomes into Lean interventions in healthcare requires a conscious effort to align environmental, social, and economic considerations with the primary goals of efficiency and quality improvement [18]. Whilst this research seeks to consider environmental sustainability in healthcare settings [96,97,98], a more comprehensive and integrated approach can contribute to a healthcare system that not only operates efficiently but also considers its long-term impact on the well-being of the community and the environment. Measuring and quantifying environmental sustainability outcomes can be complex, as they extend beyond immediate efficiency gains to include environmental impact, social responsibility, and long-term economic considerations [15]. 

The realist review outlined in this protocol will facilitate the identification of the specific contextual factors in which certain mechanisms trigger anticipated outcomes from Lean interventions [99] that include environmental sustainability outcomes in their design. We purport that the realist review will identify the available literature and case studies that facilitate further iteration of the developed IPTs and highlight where there may be instances of successful Lean projects with a focus on environmental sustainability [100] that has not been widely published or otherwise highlighted in the literature. Our study aims to improve the understanding of the inclusion of environmental sustainability outcomes in the design of Lean interventions, what works or does not work, for whom and in what contexts. We contend that this study will make an important and timely contribution with implications for healthcare policy, research and practice. 

## 5. Conclusions

This realist review protocol is designed to ensure transparency and facilitate study replication by other investigators. It begins by clearly defining research questions to focus the inquiry and establishes a comprehensive theoretical framework detailing mechanisms, contexts, and anticipated outcomes [65,77]. Detailed documentation of the search strategy, including databases, search terms, and criteria for inclusion and exclusion, ensures replicability [87]. The protocol systematically outlines data extraction and analysis procedures, including coding methods and evidence synthesis [89] and emphasizes an iterative approach allowing for continuous theory refinement and testing with emerging data [61]. Stakeholder involvement, such as reference and expert groups, are clearly emphasized for their contribution to iterative theory development [67].

The realist review outlined in this protocol will comply with the evidence-based practice for the conduct of a realist review in a systematic manner [25,59,65,69,95]. The findings of the review will facilitate an understanding of the barriers and facilitators to the inclusion of environmental sustainability goals in Lean healthcare interventions and will inform the methods that the authors use to further evaluate program theories in a realist evaluation. Whilst there is a wide body of research on Lean demonstrating its impact on effectiveness and efficiency, there is little known about the outcomes of the inclusion of environmental sustainability outcomes in Lean healthcare interventions. The realist review will provide internationally relevant findings, representing a departure from traditional systematic review methodologies and will provide a robust platform for the realist evaluation.

## Figures and Tables

**Table 1:** Using PICOC to develop Inclusion and Exclusion Criteria for the Realist Review.

PICOC Element	Key Concept	Inclusion and Exclusion Criteria
Population	Environmental sustainability goals in Lean improvement	**Included**: Healthcare organizations that have adopted Lean healthcare improvement interventions purposefully designed to achieve environmental sustainability outcomes. **Excluded**: Healthcare organizations that do not meet this inclusion criterion. **Included**: Organizations supporting, supplying or servicing healthcare settings that have adopted Lean healthcare improvement interventions purposefully designed to achieve environmental sustainability outcomes. **Included**: Non-healthcare organizations that have adopted Lean healthcare improvement interventions purposefully designed to achieve environmental sustainability outcomes. **Excluded**: Non-healthcare organizations that do not meet this inclusion criterion
Interventions	Lean and Green Lean improvement interventions purposefully designed to achieve environmental sustainability outcomes	**Included**: Lean improvement interventions purposefully designed to achieve environmental sustainability outcomes. **Excluded**: Lean improvement interventions not designed to achieve environmental sustainability outcomes. Other improvement methodologies whether or not designed to achieve environmental sustainability outcomes.
Comparison	Other QI methodologies	**Included**: Lean methodology. **Excluded**: Other Quality Improvement (QI) methodologies. **For consideration**: Whether other QI methodologies mentioned as comparators in the retrieved literature (e.g., a combined use of Lean and Six Sigma) will be included where relevant to the research questions.
Outcomes	Positive environmental impact and outcomes	**Included**: The study will look for evidence that the environmental outcomes of the Lean interventions are regularly measured as part of the monitoring of the overall impact of the improvement initiative.
	Positive impact on engagement of communities	**Included:** The study will look for evidence of how stakeholders in the settings studied have been engaged and impacted by the inclusion of environmental sustainability outcomes in Lean improvement interventions.
Context	Time	Timeframe for the consideration of studies will be from 1987 when the term Environmental sustainability began to appear in the literature.
	Language	Only studies written in or translated into English will be considered.
	Setting	Healthcare organizations, organizations delivering services to hospitals and non-healthcare organizations.

## Data Availability

Realist CPT and IPT are available as Supplementary Files.

## Supplementary Materials

The following supporting information can be downloaded at: https://www.mdpi.com/article/10.3390/ijerph21070868/s1, Supplementary File S1: Candidate Programme Theories (CPT) for Expert Panel Refinement and Supplementary File S2: Initial Programme Theories (IPT).
