# Supplementary material for: A Realist Review Protocol into the Contexts and Mechanisms That Enable the Inclusion of Environmental Sustainability Outcomes in the Design of Lean Healthcare Improvement Interventions
Source: Int J Environ Res Public Health. 2024 Jul 2;21(7):868. doi: 10.3390/ijerph21070868 (PMC11276605; doi:10.3390/ijerph21070868)
Supplement: Supplementary file 1 [file ijerph-21-00868-s001.zip › ijerph-3025459 - Supplementary File S1 - for conversion.pdf]

International Journal of
Environmental Research and Public Health

MDPI

Supplementary File S1: Candidate Programme Theories (CPT) for Expert Panel Refinement

**CPT 1**: In organisations where healthcare staff recognise the climate emergency (**C1**) and feel compelled to take local action (**C2**), and where they are supported by the organisation's leadership to take action through a workplace culture that promotes autonomy (**M1**); they can identify and make changes within their clinical practice (**O1**) that lead in aggregate to a reduction in negative impacts of their practice on climate change(**O2**) [1,2]
**CPT 2**: Where healthcare staff in an organisation which has implemented Lean methodology (**C1**), and who are trained in Lean approaches to service improvement (**C2**) are subsequently trained in carbon literacy to become more environmentally aware(**C3**), this training affords staff the time (**M1**) and provides the knowledge base (**M2**) to enable them to consider changes to their local clinical practice , resulting in an outcome of a more environmentally sustainable organisation as measured by its carbon footprint in CO2e/tonne (**O1**) [3–5]
**CPT 3**: Where healthcare organisations with sustainability teams (**C1**) whose team members have also been trained in Lean methodology (**C2**), are mandated by governments to reduce the organisation's environmental footprint (**C3**),they effectively work in an environment that is conducive to the application of their combined environmental and Lean expertise (**M1**) in support of local or national actions in which there is evidence that leads to the development of measurable outcomes to improve environmental sustainability and reduce unnecessary waste of natural resources in CO2e emissions or tonnes of waste (**O1**) [6]
**CPT 4**: In healthcare organisations where healthcare leaders have successfully introduced and embedded Lean training (**C1**) and simultaneously committed to measures to tackle the "climate emergency" (**C2**) and where environmental outcomes are considered by leaders to be part of daily work practice (**C3**) through the mechanisms of discussion at their daily huddles (**M1**) and inclusion in local Lean initiatives (**M2**) this results in clinical practices that can evidence a measurable reduction in the annual waste of natural resources (**O1**) [7]
**CPT 5**: Where Healthcare leaders committed to a Lean methodology (**C1**) adapt the approach to intentionally encompass environmental sustainability in organisational improvement activities(**C2**) and staff routinely use their knowledge and experience of environmental and sustainability methods (**M1**) to intentionally include environmental outcome metrics in designing their Lean improvement activities (**M2**), this results in the development of outcome metrics (**O1**) that quantify the measurable impact of environmental sustainability of their improvement work (**O2**) [8,9]

**CPT 6:** Within healthcare organisation where staff trained in Lean practices (**C1**) feel that their concerns around the impact of healthcare on the environment are being heard (**M1**) and addressed (**M2**) by the organisations leadership, this results in staff led local action on environmental issues (**O1**), staff who experience self-actualisation working to their full potential (**O2)** and improved staff satisfaction (**O3**) [10]
**CPT 7:** In healthcare organisations where healthcare staff who who are trained in Lean methods (**C1**) practice in tightly defined and formed teams (**C2**) are encouraged by the organisations leadership (**M1**) to use their knowledge and experience of working on improvement initiatives (**M2**) to reconsider their workplace and practice with an environmental sustainability lens (**M3**) this results in a cohesive team response to climate change (**O1**) and measurable outcomes for the reduction of measured gases, plastics and paper waste(**O2**) [11]

Reference List

1. Lee, R.; Nader, N.D. Practical environmental considerations in anesthesia practice. *Journal of clinical anesthesia* **2022**, *79*, 110522-110522, doi:10.1016/j.jclinane.2021.110522.
2. Van Demark, R.E.; Smith, V.J.S.; Fiegen, A. Lean and Green Hand Surgery. *The Journal of Hand Surgery* **2018**, *43*, 179-181, doi:10.1016/j.jhsa.2017.11.007.
3. Dües, C.M.; Tan, K.H.; Lim, M. Green as the new Lean: how to use Lean practices as a catalyst to greening your supply chain. *Journal of Cleaner Production* **2013**, *40*, 93-100, doi:10.1016/j.jclepro.2011.12.023.
4. Singh, P. Lean in healthcare organization: an opportunity for environmental sustainability. *Benchmarking : an international journal* **2019**, *26*, 205-220, doi:10.1108/BIJ-04-2018-0104.
5. Sainsbury, P.; Charlesworth, K.; Madden, L.; Capon, A.; Stewart, G.; Pencheon, D. Climate change is a health issue: what can doctors do? *Internal Medicine Journal* **2019**, *49*, 1044-1048, doi:https://doi.org/10.1111/imj.14380.
6. Organisation, W.H. *Environmentally sustainable health systems: a strategic document*; WHO Regional office for Europe: 2017.
7. Mazzocato, P.; Savage, C.; Brommels, M.; Aronsson, H.; Thor, J. Lean thinking in healthcare: a realist review of the literature. *BMJ Quality & Safety* **2010**, *19*, 376-382, doi:10.1136/qshc.2009.037986.
8. Bergmiller, G.G.; McCright, P.R. Are Lean and Green Programs Synergistic? *IIE Annual Conference. Proceedings* **2009**, 1155.
9. Pinzone, M.; Guerci, M.; Lettieri, E.; Huisingh, D. Effects of 'green' training on pro-environmental behaviors and job satisfaction: Evidence from the Italian healthcare sector. *Journal of cleaner production* **2019**, *226*, 221-232, doi:10.1016/j.jclepro.2019.04.048.
10. Hines, P. Human centred lean – introducing the people value stream. *International Journal of Lean Six Sigma* **2022**, *13*, 961-988, doi:https://doi.org/10.1108/IJLSS-03-2021-0061.

11. Burgess, N.; Currie, G.; Crump, B.; Dawson, A. *Leading change across a healthcare system : how to build improvement capability and foster a culture of continuous improvement : lessons from an evaluation of the NHS-VMI partnership*; Warwick Business School: 2022.
