# Supplementary material for: A Realist Review Protocol into the Contexts and Mechanisms That Enable the Inclusion of Environmental Sustainability Outcomes in the Design of Lean Healthcare Improvement Interventions
Source: Int J Environ Res Public Health. 2024 Jul 2;21(7):868. doi: 10.3390/ijerph21070868 (PMC11276605; doi:10.3390/ijerph21070868)
Supplement: Supplementary file 1 [file ijerph-21-00868-s001.zip › ijerph-3025459 - Supplementary File S2 - for conversion.pdf]

International Journal of
Environmental Research and Public Health

MDPI

Supplementary File S2: Initial Programme Theories (IPT)

IPT following Expert Panel Refinement

**IPT 1: Positive action** (Individual)<br><br>In organisations without an embedded Lean (quality improvement) methodology **(C1)** where healthcare staff recognise that there is a current climate emergency (**C2)** where this may be a priority issue by their employing organisation **(C3)** and where staff recognise **(M1)** the wider impact of their workplace actions and feel compelled to take personal action (**M2**), these staff can identify and make changes within their sphere of influence and practice (**O1**) that they believe will lead to a reduction in negative impacts of their work and practice on climate change (**O2**), thus contributing to the overall reduction of the carbon footprint of the organisation **(O3)** [1,2]
**IPT 2: Basic training** (Consider if this training is genuinely available to all or a small subset of staff)<br><br>Where healthcare staff in an organisation which has implemented Lean (quality improvement) methodology (**C1**), are trained in Lean approaches to service improvement (**C2**) and are subsequently trained in basic carbon literacy to become more environmentally aware (**C3**) the carbon literacy training affords staff a knowledge base (**M1**) and generates enthusiasm (**M2**) to enable them to consider the wider impact of changes to their work practice resulting in a change in their own attitude and behaviour towards waste **(O1)** and actions that contribute to a more environmentally sustainable organisation **(O2)**, seeking support to understand and measure the process carbon footprint in CO2e/tonne (**O3**) [3–5]
**IPT 3: Policy implementation Institutional setting** (Where Lean and green initiatives may not be connected)<br><br>Where healthcare organisations that use Lean (quality improvement) as an improvement approach (**C1**) are mandated by government legislation or local Boards to reduce carbon emissions and the organisational environmental footprint (**C2**) within a specified timeframe **(C3)** they move to promote this mandate through the mechanism of developed dedicated sustainability teams with expert environmental knowledge **(M1)** that effectively work in an environment that is conducive to the application of this knowledge to a healthcare setting **(M2)** in support of local or national evidenced initiatives **(M3)** resulting in the development of actions **(O1)** that will lead to measurable outcomes to improve environmental sustainability and a reduction of the waste of natural resources in CO2e emissions or tonnes of waste (**O2**) to meet, for example a target of Net Zero in the NHS by 2040 **(O3) [6]**

**IPT 4: Lean and Green synergy of approaches** (Wider infrastructural setting)<br><br>In healthcare organisations where healthcare leaders have successfully introduced and embedded Lean (quality improvement) methods and training (**C1**) and simultaneously and publicly committed to measures to tackle the “climate emergency” (**C2**) and where environmental outcomes are considered by leaders to be part of daily work (**C3**) staff routinely use their knowledge and experience of environmental and sustainability methods **(M1)** to consider wider environmental elements of waste as part of their Lean practice **(M2)**, through the mechanisms of discussion at their daily huddles (**M3**) and inclusion of metrics in local Lean initiatives (**M4**) resulting in the development of outcome metrics (**O1**) that quantify the measurable impact of environmental sustainability of their improvement work (**O2**) changes in processes and practices that can evidence a measurable reduction in the annual waste of natural resources (**O3**) that can contribute to the overall reduction in the carbon footprint of the organisation **(O4)** [7]
**IPT 5: Teamwork** (Cross-disciplinary connectedness)<br><br>Within healthcare organisations where staff trained in Lean (quality improvement) practices **(C1)** purposefully develop a positive and inclusive narrative around climate change **(C2)** linked to the values and mission of the organisation **(C3)** to explain and contextualise the impact of their healthcare activity on the environment **(C4),** the facilitated development of this understanding of the link between values, mission and environmental goals **(M1),** and organisational recognition of staffs’ environmental concerns (**M2**) and staffs’ confidence that these concerns are being heard and addressed by the leadership of the organisation **(M3),** and there is a common understanding of these concerns them within the context of wider social and economic benefits across the organisation **(M4),** this results in staff pride and commitment to the action of their employing organisation **(O1)** encouraging local action on environmental issues (**O2**), and encourages positive behaviours of staff as ambassadors of this effort to the wider community **(O3)** [8,9]
**IPT 6: Staff Wellbeing** (Autonomy of practice)<br><br>Where healthcare organisations use their commitment and experience of use of a Lean (quality improvement) methodology **(C1)**<br>to allow staff the autonomy to recraft their roles and tasks to include more environmentally sustainable activities **(M1)** as part of an established change methodology to remove waste from an existing process (M2), this results in a staff experiencing a sense of self-actualisation **(O1)** working to their full potential (**O2)** and a positive impact from their contribution to the climate change effort **(O3)** resulting in improved staff satisfaction (**O4**) [10]

**IPT 7: Teamwork** (Local tightly formed teams that behave as discrete entities within organisations)<br><br>In healthcare organisations where healthcare staff who are trained in Lean methods (**C1**) practice in tightly defined and formed teams (**C2**) and where they are encouraged by the organisations' leadership (**M1**) to use their knowledge and experience of working on improvement initiatives (**M2**) to reconsider their workplace and practice with an environmental sustainability lens (**M3**) this results in a localised and cohesive team response to climate change (**O1**) and measurable outcomes for the reduction of local material waste and changes in practice which can be measured and shared (**O2**) [11]
**IPT 8: Teamwork** Community to practice<br><br>Where healthcare organisations intentionally link the inter-relationship between their embedded Lean (quality improvement) effort and all three pillars of sustainability **(C1)** to develop an understanding of social responsibility, financial viability and environmental protection through the branding **(M1)** of joint initiatives and active support **(M2)** of informal networks to discuss common causes which are of significant importance to staff **(M3)** this results in the development of staff driven and local initiatives **(O1)** that can be celebrated **(O2)** and may, dependant upon the initiative, have a measurable impact on the carbon footprint of the organisation **(O3)** [12]

Summary of Initial programme theories

**IPT 1**	**Positive individual action**	**C3 M2 O4**
**IPT 2**	**Impact of basic training**	**C3 M2 O3**
**IPT 3**	**Policy implementation**	**C3 M3 O4**
**IPT 4**	**Synergy of approaches**	**C3 M4 O4**
**IPT 5**	**Teamwork: Cross-disciplinary connectedness**	**C4 M4 O3**
**IPT 6**	**Staff wellbeing**	**C1 M2 O4**
**IPT 7**	**Teamwork: Local tightly connected teams**	**C2 M3 O2**
**IPT 8**	**Teamwork: Communities of practice**	**C1 M3 O3**

Reference List

1. Lee, R.; Nader, N.D. Practical environmental considerations in anesthesia practice. *Journal of clinical anesthesia* **2022**, *79*, 110522-110522, doi:10.1016/j.jclinane.2021.110522.
2. Van Demark, R.E.; Smith, V.J.S.; Fiegen, A. Lean and Green Hand Surgery. *The Journal of Hand Surgery* **2018**, *43*, 179-181, doi:10.1016/j.jhsa.2017.11.007.
3. Dües, C.M.; Tan, K.H.; Lim, M. Green as the new Lean: how to use Lean practices as a catalyst to greening your supply chain. *Journal of Cleaner Production* **2013**, *40*, 93-100, doi:10.1016/j.jclepro.2011.12.023.
4. Singh, P. Lean in healthcare organization: an opportunity for environmental sustainability. *Benchmarking : an international journal* **2019**, *26*, 205-220, doi:10.1108/BIJ-04-2018-0104.
5. Sainsbury, P.; Charlesworth, K.; Madden, L.; Capon, A.; Stewart, G.; Pencheon, D. Climate change is a health issue: what can doctors do? *Internal Medicine Journal* **2019**, *49*, 1044-1048, doi:https://doi.org/10.1111/imj.14380.
6. Organisation, W.H. *Environmentally sustainable health systems: a strategic document*; WHO Regional office for Europe: 2017.
7. Mazzocato, P.; Savage, C.; Brommels, M.; Aronsson, H.; Thor, J. Lean thinking in healthcare: a realist review of the literature. *BMJ Quality & Safety* **2010**, *19*, 376-382, doi:10.1136/qshc.2009.037986.
8. Bergmiller, G.G.; McCright, P.R. Are Lean and Green Programs Synergistic? *IIE Annual Conference. Proceedings* **2009**, 1155.
9. Pinzone, M.; Guerci, M.; Lettieri, E.; Huisingh, D. Effects of 'green' training on pro-environmental behaviors and job satisfaction: Evidence from the Italian healthcare sector. *Journal of cleaner production* **2019**, *226*, 221-232, doi:10.1016/j.jclepro.2019.04.048.

10. Hines, P. Human centred lean – introducing the people value stream. *International Journal of Lean Six Sigma* **2022**, *13*, 961-988, doi:https://doi.org/10.1108/IJLSS-03-2021-0061.
11. Burgess, N.; Currie, G.; Crump, B.; Dawson, A. *Leading change across a healthcare system : how to build improvement capability and foster a culture of continuous improvement : lessons from an evaluation of the NHS-VMI partnership*; Warwick Business School: 2022.
12. Ward, M.E.; Daly, A.; McNamara, M.; Garvey, S.; Teeling, S.P. A Case Study of a Whole System Approach to Improvement in an Acute Hospital Setting. *Int J Environ Res Public Health* **2022**, *19*, doi:10.3390/ijerph19031246.
